# An Approach to Identify “Right” Problems for Initial Quality Improvement Projects

**DOI:** 10.4103/JQSH.JQSH_16_19

**Published:** 2020-01-27

**Authors:** Sergio R. Gutiérrez Ubeda

**Affiliations:** Center of Investigation and Study of Health, National Autonomous University of Nicaragua, Managua, Nicaragua

## Introduction

Since the 1950s to date, first in Japan and then in other countries worldwide, a growing number of organizations have been promoting project-based quality improvement (QI) methodologies. This trend has been part of a response to the higher expectation of quality of virtually “everything” required in the life of individuals and societies.

A QI project is “a chronic problem scheduled for solution.”[1] A QI project follows a QI methodology, which, briefly explained, consists of selecting a problem, describing its related process, diagnosing causes, and seeking solutions of problems through process improvement.[2]

One critical factor for successful implementation of QI projects is selecting one problem of the “right” type for QI methodology. However, a poor selection and prioritization of problems of QI projects remain among their most common critical failure factors.[3]

Project teams might wrongly select problems for QI projects partly because teaching materials (e.g., books, manuals, and articles) and training courses on QI project methodologies ignore or barely mention what are and how to identify “right” problems for such projects.

The objective of this article was to propose a semi-structured approach toward problem identification for QI projects. This approach aimed to help QI project teams conducting their initial QI projects, especially in contexts that lack expertise and information systems to identify QI problems of objects of team interest. Objects of interest for QI project teams might be tangible things such as equipment and instruments; intangible entities such as knowledge and culture; and entities that might have both tangible and intangible components such as systems, processes, and services.[3,4]

## An Approach toward Problem Identification for Quality Improvement Projects

The approach toward problem identification for QI projects recommended clarifying a sequential set of six questions while project teams are searching for quality problems for QI projects. I will explain for each of the six questions, its purposes, expected outputs, and examples of outputs.

### What are the possible objects of interest for the quality improvement project?

In this step, project teams have to know a list of possible objects of interest for QI project. Such list would show team awareness of many possible objects under their responsibility, some of which might be or not be good targets of QI projects. With this list, project teams would try to select objects that are best targets for QI projects from the start of their projects, instead of choosing the first object that comes to mind without awareness of other possibilities, a practice that is not rare to observe in real settings. Good targets are objects with features that increase their possibilities of having “right” quality problems for QI projects. Examples of such features include processes that are well structured, repetitive, with high frequency of quality problems, and controllable.[5] Well-structured processes have explicit, detailed, and standardized instructions, rules, resources, activities, and procedures that allow them to perform in the same manner repeatedly. Well-structured processes, especially together with standardized inputs, would be able to produce more predictable results than less-structured processes. In other words, the linkage between changes in processes and changes in results is higher. In this type of processes, it is easier for projects teams to prove whether project solutions were effective.

Frequently produced processes are more suitable for typical statistical analysis conducted in QI projects because they produce more data than less frequently produced processes. Basic, and sometimes, advanced statistical analysis would have stronger statistical power when the amount of data matches requirement of statistical tools such as histogram (e.g., 40 or more values) and control chart (e.g., 20–30 values).[6]

Processes with frequent problems would require project teams to collect less data to detect such problems and to monitor their behavior before and after project intervention. With rare problems, projects need to collect large amounts of data or to perform complex statistical analysis. In such projects, the unavailability of resources such as budget and statistical expertise would compromise project feasibility and success.

Last but not the least, the process of interest should be controllable, either partially or totally, within a project team power. If project teams have no power or resources to implement all required actions of QI project methodologies, it means the project is not feasible.

### What is the object of interest for the quality improvement project?

Assuming that a project team has already selected one object with suitable features for QI projects, the next step is to clarify what the unit of the selected object is. Such clarification is important to ensure that all interested stakeholders of QI projects (e.g., project team members) are aligning their effort in the same direction toward the same object. Sometimes, an object unit is by design identified through a distinctive name and model such as manufactured goods, but this is not a practical approach to all types of objects (e.g., services). Without a clear identification of an object unit, it might be mistaken with another object. In such cases, providing a name and operational definition of the unit might be minimal requirements to avoid such confusion. On one hand, a name or label might describe distinctive features of the object of interest. Poorly labeled objects might lead to confusion between similar objects. For example, if a team selected a package of services labeled as “prenatal care,” it might be a broad label for identifying and defining such service as a particular unit. A prenatal care might have different versions depending if it is for low- or high-risk pregnant woman, and if the delivery of prenatal care is during a first or subsequent visit to a health-care facility. Each of such versions has its distinguishing components, features, and problems; each of which needs to be controlled following their particularities. A more precise name instead of “prenatal care” might be for one of its versions “prenatal care for low-risk pregnancy during first visit to clinic.”

On the other hand, a definition of the unit of object of interest aims to describe the object in more detail and to highlight its distinctive features, deeper than its name. A definition of an object might describe the nature of the unit as a whole with its component parts and interrelations. The precision of delimitation of boundaries and a detailed description of the object of interest would help to determine what the object it is and it is not. The amount of detail for describing an object would be enough until it is unlikely that some interested stakeholders would confuse an object of interest with other objects.

### What are the quality characteristics of the object?

In this step, project teams have to develop measurable characteristics of quality of the object of interest for the QI project. A definition of quality of objects would provide the QI project team with a guide to identify quality indicators, to measure them and to assess whether an object is of good quality or not. To define the quality of an object, there is a need to know what quality is. There are many definitions of quality.[7] For example, Garvin[8] grouped product quality definitions into five types: transcendent, user based, product based, value based, and manufacturing based. Also, the health-care quality definition by the United States Institute of Medicine (IOM) is widely quoted as “The degree to which health services for individuals and populations increase the likelihood of desired health outcomes and are consistent with current professional knowledge.” Finally, there is a growing acknowledgment that quality health services should be effective, safe, and people centered.[7]

To define the quality of an object, this article recommended using an operational definition of quality. Such definition describes characteristics that are critical to quality called “quality characteristics”[4,9] or CTQ for short.[10] Recently in health care, the label “key quality characteristic”[11] has the same meaning of CTQ. Since the 1950s, the Japanese manufacturing industries have used with success the CTQ concept,[9] and recently, its use is growing in the health-care industry as well as other industries and countries. A quality characteristic is an inherent characteristic of a good or service, process, or system related to a requirement. Inherent means “existing in something, especially as a permanent characteristic” and requirement means “need or expectation that is stated, generally implied or obligatory.”[4] Objects might have a high number of characteristics; the more the complexity of the object the more the number of characteristics it might have. The characteristics that should be preferred as CTQs are by definition those directly related to customer requirements, CTQs are also called “true quality characteristics.”[9] However, when measuring true CTQs is not possible, a project team has to identify and measure substitute CTQs and indicators. For a detailed discussion on true and substitute CTQs and for the CTQ use in health-care context, the reader can check Ishikawa's book[9] and Lloyd's book, respectively.[11] Table 1 shows examples of quality characteristics of different types of objects.

**Table 1:** Examples of quality characteristics of four types of objects

**Food of a restaurant**	**Scientific knowledge**	**Data**	**Healthcare service**
Safety	Original	Believable	Safety
Taste	Relevant	Relevant	Effective
Smell	Rigorous	Accurate	Patient centered
Amount	Generalizable	Interpretable	Timely
Appetizing	Ethical	Objective	Efficient
Temperature	Communicable	Completeness	Aesthetics

Two challenges while defining the quality of objects are achieving comprehensiveness and establishing a priority order of hierarchy of CTQs. Comprehensiveness here means identifying all CTQs that are the best representative of quality of the object of interest. CTQs should be carefully determined. When a list of CTQs of objects of interest is not available, a project team has to build one. Different approaches to build such list might include using quality tools such as brainstorming and/or conducting qualitative research and benchmarking established dimensions of quality, such as the IOM dimensions of health-care quality (e.g., safe, effective, patient centered, timely, efficient, and equitable).[7]

Making a priority order of several CTQs of objects is necessary when it is not possible to control all CTQs for reasons such as insufficient resources and lack of technology. In such cases, a good practice would be establishing a set of criteria for prioritizing what CTQs are best candidates for control. Examples of criteria for prioritizing CTQs include safety and relevance for costumers.

### What are the quality indicators that measure the quality characteristics of the object?

Controlling CTQ behavior requires developing and measuring indicators.[11] In this step, project teams have to develop a list of indicators that best measure the behavior of CTQs of the object of interest for the QI project. Evidence-based indicators should be preferred if available. Otherwise, establishing criteria for selecting good indicators is advisable. Examples of criteria of good-quality indicators include validity, reliability, relevance, feedback time frame, and feasibility of data collection.[7] This article recommended selecting indicators that fulfill a set of criteria for good-quality indicators. It is not recommendable selecting CTQ indicators based on only one criterion, such as indicators that are easy to measure or that their data are already collected through routine information systems, because they might lead to biased results on interpreting the state of quality of objects.

### What are the quality problems of the object of interest?

**Figure 1:**
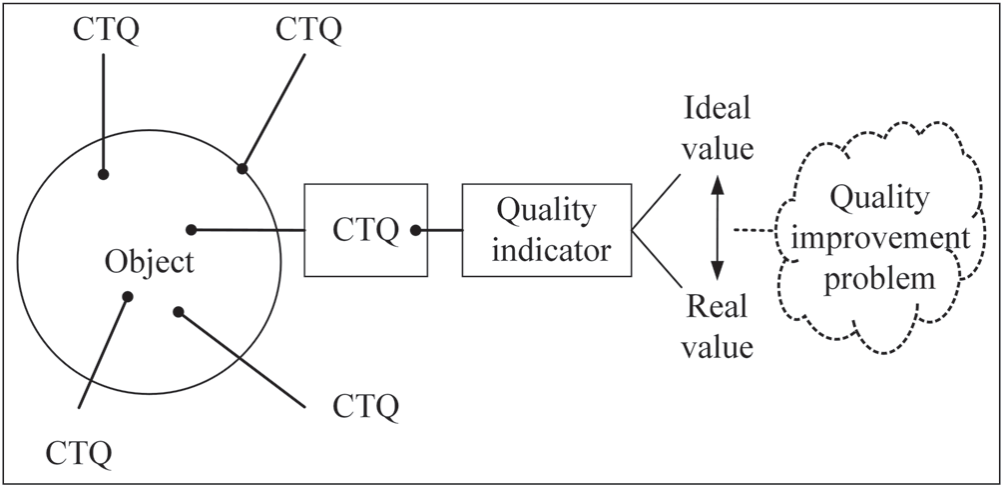
Schematic relationship between object, characteristic that is critical to quality, quality indicator, and quality improvement problem. CTQ = characteristic that is critical to quality

In this step, project teams have to develop a list of quality problems of the object of interest for QI project. The *Cambridge Dictionary* defines problem as “a situation that causes difficulties,” “a matter about which it is difficult to decide what to do,” and “a question to be answered or solved.”[12] This article defined quality problems as gaps between current and desired state of CTQs as measured by quality indicators. For example, a door-to-needle time for intravenous thrombolysis in acute ischemic stroke might be in normal operational conditions of 60 min or less. Every time such door-to-needle time is more than 60 min, such gap represents a quality problem. Figure 1 shows a schematic representation of the object of interest, its quality characteristics, quality indicators, and QI problems. Table 2 shows one example of a health-care service unit (e.g., appendectomy) with examples of its quality characteristics, quality indicators, and quality problems.

**Table 2:** Examples of characteristics that are critical to quality, quality indicators, and quality problems of an appendectomy service

**CTQ**	**Quality indicators**	**Quality problem**
Safety	Number of sterile instruments	Contaminated instruments
	Clean surgical wound	Infected surgical wound
Time	Time length of procedure in minutes	Prolonged surgical time
	Postsurgical recovery time in days	Prolonged postsurgical recovery time
Effectiveness	Inflamed appendix completely removed	Accidental injury during removal of appendix
Aesthetics	Length of scar of 5–7 cm	Length of scar of more than 7 cm

CTQ = characteristic that is critical to quality

### Is the problem suited for a quality improvement project?

In this step, project teams have to assess suitability of quality problems for QI project methodology. There are many types of quality problems and their solutions do not always require a QI project methodology. Simple quality problems might require “just do it” actions to solve them. Complex quality problems might require complex solutions such as conducting scientific research projects or developing new technologies. There is not an assessment tool of problems to be sure by 100% whether a problem is suited for a new QI project. However, assessing features that characterize QI problems [Table 3] might help toward this end. The project team would apply a matrix for problem selection, built with such criteria, to the list of problems they completed before. If objective quality data to assess such characteristics are not available, then a team needs to prepare and perform a data collection plan. If during a training course of QI methodology, there is no time to collect such data, as it is usually the case, a project team might perform a preliminary subjective assessment of problems. This preliminary assessment might consist of scoring problems based on subjective opinions of project team members.

**Table 3:** Features of “right” problems for quality improvement projects

**Features**	**Description**
Relevance	Important for customers
Team priority	The project team members have roles in the selected process
Time frame	The project team might solve the problem between 3 and 6 months
Evidence based	It is feasible to collect reliable data to measure the problem behavior
Frequent	It is possible to observe the behavior of the problem with frequently repeated measurements (e.g., daily measurement)
Stable	A measured problem behaves within agreed limits of statistical control. Well-structured and controlled processes produce more stable results and problems
Chronic	A problem is persistent along measurements in different process cycles of existing operating processes. The process might be stable or unstable
Unknown causes	The causes of the problem are not readily apparent. An investigation is required for their disclosure
Unknown solutions	Solutions for the problem are not obvious. Possible solutions might be in mind but without proof of their effectiveness
Proper size	One or few CTQs of one or few objects
Controllable	The project team might influence the behavior of the problem with project team available resources

CTQ = characteristic that is critical to quality

Finally, a project team that is aware of “wrong” types of problems for QI projects [Table 4] might help the process of identifying the “right” types.

**Table 4:** Examples of “wrong” problems for quality improvement projects

**Type**	**Examples**
Too big	Problems that include multiple objects, multiple locations of one or more objects, and multiple levels of one or more objects
Too small	Problems that are “causes” of a CTQ problem, and one-person problem
Too complex	Too sophisticated problem, such as when a problem complexity overpasses the level of expertise that possesses or is accessible to project team sponsors and members
Unstable	Unstable problem means that its behavior is outside of agreed limits of statistical control.
	Unstable problems usually require quality control methodologies for their solution
Mismatch problem	The problem selected for a quality improvement project is not quality driven, such as access and cost-driven problems
Poor evidence	Poor supportive data, such as a problem disclosed with static data (e.g., mean) alone
Infrequent problem	Low frequency problems that are difficult to detect even with large sample sizes

CTQ = characteristic that is critical to quality

## Conclusion

This article presents a semi-structured approach to identify “right” types of quality problems for QI projects. This approach recommended clarifying a sequential set of six questions while project teams are searching for quality problems for QI projects. Clarification of these questions might ensure that all interested stakeholders of QI projects (e.g., project team members), from the start of such projects, are aligning their effort toward solving a methodological sound and worthwhile quality problem. This article might help complementing teaching materials and strengthening training efforts on methodologies for conducting QI projects, particularly in the process of identification of quality problems.
